# A Gut Feeling: Acute Liver Failure – An Unusual Manifestation of Malignant Catatonia

**DOI:** 10.7759/cureus.15242

**Published:** 2021-05-25

**Authors:** Sara Kiparizoska, Willam Davis, Michelle Duong, Nayrana Griffith, Christine Hsu

**Affiliations:** 1 Internal Medicine, Georgetown University, Washington DC, USA; 2 Medstar Georgetown Transplant Institute, Georgetown University, Washington DC, USA

**Keywords:** acute liver failure, catatonia, psychosis, neuroleptic malignant syndrome, alf, nms

## Abstract

We present a rare case in which malignant catatonia led to acute liver failure (ALF). A 19-year-old male was admitted for psychosis and developed ALF with a peak aspartate aminotransferase and alanine aminotransferase of 5,728 U/L and 7,735 U/L, respectively, and a peak international normalized ratio of 7.1. Liver biopsy showed significant confluent necrosis involving >70% of the liver tissue. He was listed for a liver transplant but was ultimately taken off of because of significant improvement with treatment by N-acetylcysteine infusion. Through our research, we found that symptoms of hepatitis can be seen with psychotic disorders, but ALF is rare.

## Introduction

Malignant catatonia is a life-threatening neuropsychiatric syndrome that can manifest with symptoms of autonomic dysregulation, delirium, rigidity, and fever in people with an underlying psychiatric history. Neuroleptic malignant syndrome (NMS) also has a similar clinical presentation but is associated with the use of antipsychotic agents. In both disorders, particular symptoms of hepatitis can be seen, but cases of acute liver failure (ALF) are rare. We present a rare case in which malignant catatonia led to ALF. To our knowledge, there are no other cases reporting this condition.

## Case presentation

A 19-year-old male with a history of recently diagnosed schizoaffective disorder was admitted for altered mental status, rigidity, and fevers. He was diagnosed with schizoaffective disorder nine months prior to presentation, and was initially started on risperidone but switched to aripiprazole and then olanzapine. His presentation was concerning for NMS and treatment was started with bromocriptine along with avoidance of all dopaminergic antagonists. His liver enzymes were initially mildly elevated with aspartate aminotransferase (AST) 138 U/L, alanine aminotransferase (ALT) 74 U/L, alkaline phosphatase 102 U/L, international normalized ratio (INR) 1.2, total bilirubin 1, and creatinine kinase (CK) 728 U/L. Due to severe agitation, he was administered haloperidol, and on the following days, he was observed to develop significantly rising liver enzymes and worsening liver synthetic function. His AST and ALT peaked at 5,728 U/L and 7,735 U/L, and his INR peaked at 7.1 prior to transfer to our facility. The patient was started on N-acetylcysteine (NAC) infusion and transferred to our center for liver transplant evaluation.

On arrival, physical examination was significant for tachycardia to 199 beats per minute, hyperthermia to 38.6°C, and rigidity of all extremities. The patient was somnolent but oriented and answered all questions appropriately. His abdomen was soft and nontender, and he did not have any asterixis. Other labs were significant for CK 15,293 U/L, lactic acid 3.6 mmol/L, and white blood cell count 15.7 k/uL.

**Figure 1:**
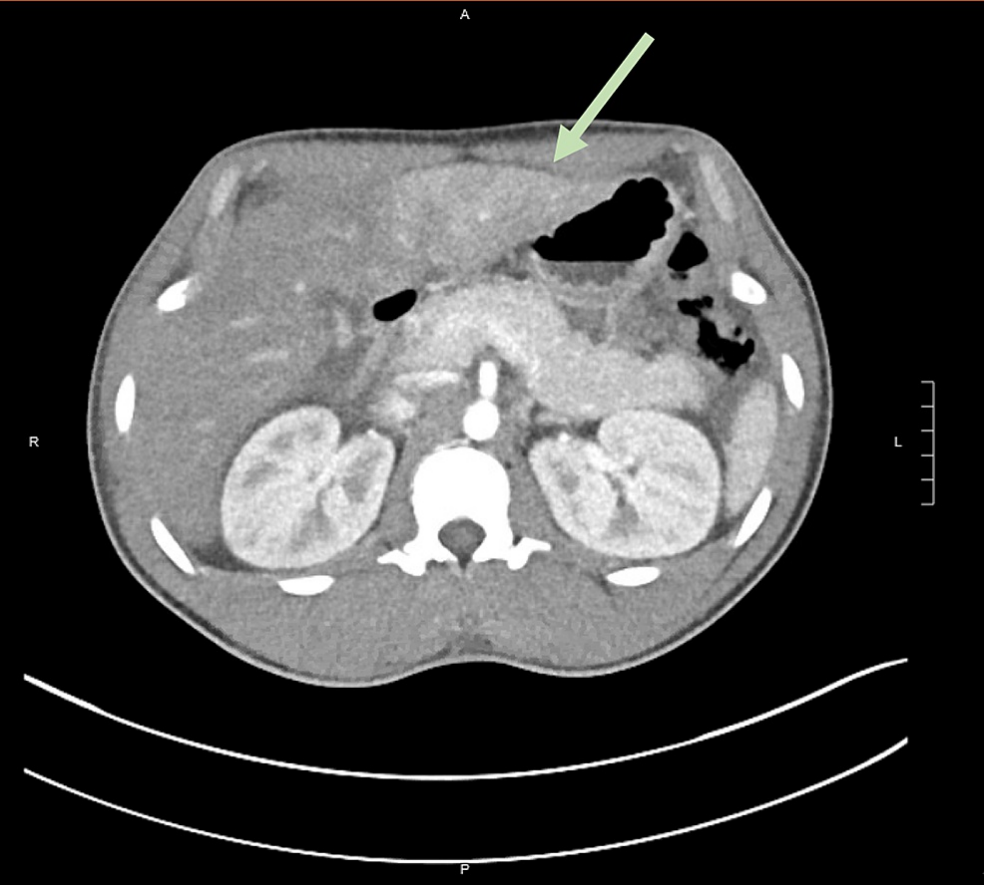
Abdominal CT scan with contrast in the arterial phase showing slight heterogeneous left lateral liver lobe enhancement (green arrow). Overall, the liver is normal in morphology and contour. CT: computed tomography

During hospitalization, the patient underwent an extensive workup to exclude alternative causes of ALF. His serologies were negative for viral hepatitis (A, B, C), human immunodeficiency virus, cytomegalovirus, Epstein- Barr virus, and herpes simplex virus. Autoimmune serologies, such as antinuclear antibodies (ANA), F-actin, liver kidney microsomal type 1, antimitochondrial immunoglobulin G (IgG), and serum quantitative IgG were within normal limits. He had normal serum ceruloplasmin and serum copper levels. All drug and toxin screens were negative and specifically included acetaminophen, cocaine, phenylcyclohexyl piperidine, amphetamines, and heavy metals. Abdominal computed tomography (CT) was significant for a normal-sized liver with slight heterogeneous enhancement, likely perfusional changes. Overall, normal liver morphology and contour were noted. There was no splenic or biliary pathology (Figure 1).

**Figure 2:**
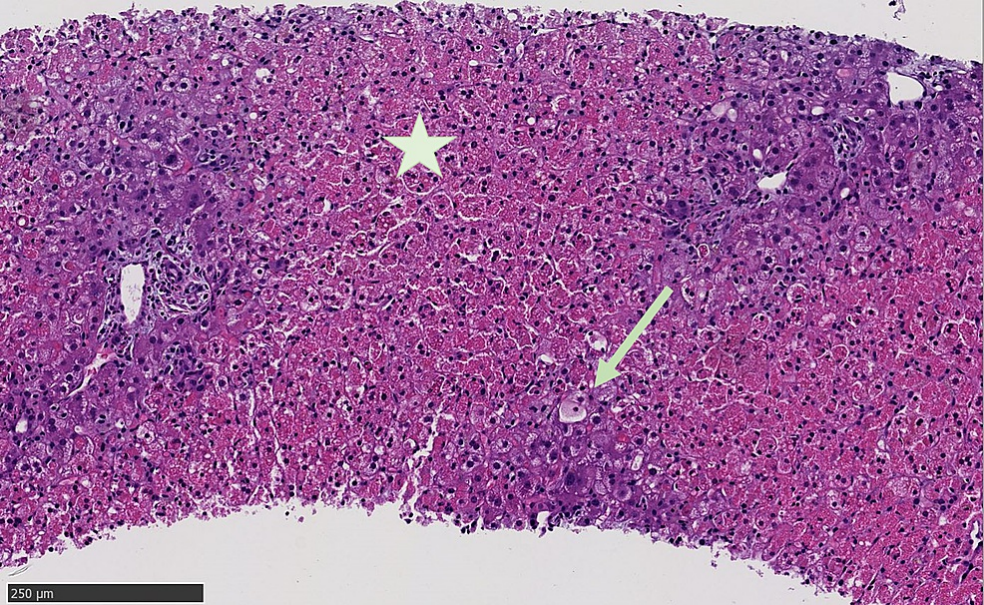
Core liver biopsy, 10× magnified. Greater than 70% of the parenchyma shows liver necrosis (light pink area marked by the star). The arrow represents nonviable ghost hepatocytes.

Finally, the patient underwent a transjugular liver biopsy. Pathology showed preserved lobular architecture with significant confluent necrosis most prominent in zone two and three involving >70% of the liver tissue. Trichrome stain confirmed the early collapse of reticulin framework seen in necrosis but only with mild sinusoidal fibrosis and no bridging (stage 1) (Figure 2). Scattered hepatocytes showed karyorrhexis but without significant inflammation. Bile ducts were intact with no inflammation. Iron and copper stains were negative. Given the liver biopsy findings, the patient was listed for liver transplant as status 1a. Additionally, for the ongoing fevers and poor mental status, the patient also underwent lumbar puncture with cerebrospinal fluid studies that were negative for infectious and paraneoplastic etiologies.

Over the course of the hospitalization, the patient was treated with NAC therapy to treat nonacetaminophen ALF and bromocriptine for the initially suspected NMS. Together with psychiatry, we decided not to pursue treatment with dantrolene for NMS given dantrolene’s hepatotoxicity profile. As the patient had not received any antipsychotics since admission, the initial diagnosis of NMS was replaced with malignant catatonia as the underlying neuropsychiatric disease causing ALF. Liver enzymes and CK intermittently continued to rise in correlation with episodic rigidity and psychotic episodes. Overall, he was ultimately taken off of the transplant list for significant improvement. Liver enzymes were close to normal values 53 days after admission.

## Discussion

**Figure 3:**
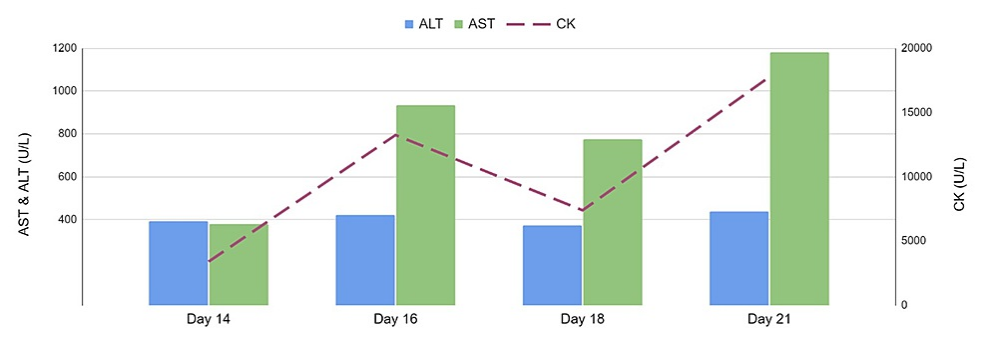
Fluctuations in liver enzymes and CK correlating with patient’s psychotic episodes. AST: aspartate aminotransferase; ALT: alanine aminotransferase; CK: creatine kinase

Malignant catatonia is the most likely cause of this patient’s ALF. A life-threatening subtype of catatonia, malignant catatonia can have fulminant progression and includes fever, autonomic instability, delirium, and rigidity [1]. Reports of organ involvement with malignant catatonia include acute renal failure, cardiac arrest, respiratory failure, and hepatocellular damage [2]. To our knowledge, there have been cases of catatonia after liver transplantation and upon initial diagnosis of Wilson’s disease, but there have been no presentations of catatonia as the cause of ALF [3,4]. As NMS was also on our initial differential, we did find a few NMS and ALF case reports [5]. However, NMS was excluded from the underlying diagnosis as the patient did not receive any antipsychotic medications for over a month during his hospitalization but continued to show fluctuations in liver enzymes and CK that correlated with his psychotic episodes (Figure 3). However, it is important to note that some psychopathologists view catatonia as a syndrome and have even suggested that malignant catatonia and NMS be merged as a similar condition, the latter being a medication-related variant of malignant catatonia [6].

Broadly speaking about ALF, studies show that in the United States, 14% of cases had indeterminate causes [7]. Regarding our case, there is limited literature delineating the relationship between psychiatric disease and ALF. The prevalence of liver disease in patients with psychiatric illness is unknown. However, those with severe psychiatric illness are at a higher risk for metabolic syndromes such as liver disease [8]. In our case, we successfully treated the patient with NAC for nonacetaminophen ALF along with control of the underlying psychiatric symptoms [9].

## Conclusions

The main learning point from this unusual case is the importance of evaluating liver function in patients with atypical psychiatric presentations. Along with the formation of a multidisciplinary team, increased awareness about ALF and psychiatric disease may improve outcomes by faster initiation of treatment and a decrease in the need for liver transplants.
